# Supplementary material for: On the importance of time scales when studying adaptive evolution
Source: Evol Lett. 2018 Oct 24;3(3):240–7. doi: 10.1002/evl3.86 (PMC6546376; doi:10.1002/evl3.86)
Supplement: Supplementary file 1 [file EVL3-3-240-s001.docx]

**Supplementary material – Perrier & Charmantier – On the importance of time scales when studying adaptive evolution**

Supplementary material 1. Reanalysis of the COL4A5 region. We downloaded the SNP dataset from Bosse et al. 2017 and used vcftools (Danecek et al. 2011) to estimate LD across the genome for the entire dataset. We then estimated LD for both populations separately along the chromosome 4A. To represent long distance LD, we kept LD values between each pairs of markers distant from 20kb to 200kb. We smoothed these statistics using the loess function native to R. We estimated F_ST_ using Plink (Purcell et al. 2007). We performed eigenGWAS tests (Chen et al. 2017) in the UK, the NL and both. EigenGWAS were performed in chromosome 4A only and not on the entire genome, in order to be more comparable with the simulation outputs on one chromosome. Results from whole genome eigenGWAS can be found in figure S2 in Bosse et al. (2017).

Supplementary material 2. Simulation of a chromosome similar in size to the chromosome 4A in great tit, and containing a region of reduced recombination of approximately the same size as the COL4A5 region. We used SLiM (Haller et al. 2017) to simulate a very simple scenario of reduced recombination in a 1.6Mb region within a 20Mb long chromosome. Recombination rate was equal to 1e-5 from 0 to 11.5Kb and from 13.1Kb to 20Mb. Recombination rate was equal to 1e-100 11.5Kb to 13.1Kb. Population size was equal to 1000. Simulation lasted 2000 generations. Mutation rate was 1e-7. We implemented 3 types of mutations: i) neutral, 50% of the mutations, ii) slightly deleterious (fitness effect of -0.1), 25% of the mutations, iii) severely deleterious (fitness effect of -0.9), 25% of the mutations. We exported a VCF dataset containing about 8500 SNPs from a randomly chosen simulation. We used vcftools to estimate LD across the chromosomes and nucleotide diversity (PI) over 200 kb windows. To represent long distance LD, we kept LD values between each pairs of markers distant from 20kb to 200kb. We performed eigenGWAS tests (Chen et al. 2017). We estimated iES (Sabesti et al. 2007) using the R package rehh (Gautier et al. 2016). We smoothed statistics using the loess function native to R.
